# Deep Sequencing of Hepatitis B Virus Reveals Clinically Relevant Low-Frequency Variants Among People Living with HIV in Botswana

**DOI:** 10.3390/v18080904

**Published:** 2026-08-17

**Authors:** Tsholofelo Sethibe, Wonderful Tatenda Choga, Florence G. Gaongalelwe, Bonolo B. Phinius, Gorata G. A. Mpebe, Kabo Baruti, Chanana Dorcus Tsayang, Goabaone Mbae, Basetsana Katlo S. Phakedi, Patience Motshosi, Linda Mpofu-Dobo, Mosimanegape Jongman, Sikhulile Moyo, Motswedi Anderson, Simani Gaseitsiwe

**Affiliations:** 1 Botswana Harvard Health Partnership, Gaborone Private Bag BO320, Botswana; tratsoma@bhp.org.bw (T.S.); wchoga@bhp.org.bw (W.T.C.); fgaongalelwe@bhp.org.bw (F.G.G.); bphinius@bhp.org.bw (B.B.P.); gmpebe@bhp.org.bw (G.G.A.M.); barutik@ub.ac.bw (K.B.); ctsayang@bhp.org.bw (C.D.T.); gmbae@bhp.org.bw (G.M.); bphakedi@bhp.org.bw (B.K.S.P.); pmotshosi@bhp.org.bw (P.M.); ldobo@bhp.org.bw (L.M.-D.); jongmanm@ub.ac.bw (M.J.); manderson@bhp.org.bw (M.A.); 2 Department of Biological Sciences, School of Physical and Life Science, Faculty of Natural and Applied Science, University of Botswana, Gaborone Private Bag UB0022, Botswana; 3 Division of Medical Virology, Microbiology Unit, Faculty of Medicine and Health Sciences, University of Zimbabwe, Harare P.O. Box MP167, Zimbabwe; 4 Department of Agricultural Genetics and Cell Technology, Faculty of Agriculture and Technology, National University of Science and Technology, Bulawayo P.O. Box AC 939, Zimbabwe; 5 Okavango Research Institute, University of Botswana, Maun Private Bag 285, Botswana; 6 School of Allied Health Professions, Faculty of Medicine and Health Sciences, University of Botswana, Gaborone Private Bag UB0022, Botswana; 7 Department of Biological Sciences and Biotechnology, School of Life Sciences, Botswana International University of Science and Technology, Palapye Private Bag 16, Botswana; 8 Department of Applied Biology and Biochemistry, National University of Science and Technology, Bulawayo P.O. Box AC 939, Zimbabwe; 9 Department of Immunology and Infectious Diseases, Harvard T.H. Chan School of Public Health, Boston, MA 02115, USA; 10 School of Health Systems and Public Health, University of Pretoria, Private Bag X20, Pretoria 0028, South Africa; 11 The Francis Crick Institute, 1 Midland Road, London NW1 1AT, UK; 12 Africa Health Research Institute, Private Bag X7, Congella, Durban 4013, South Africa

**Keywords:** hepatitis B virus, diversity, minority variants, deep sequencing

## Abstract

(1) Background: The Hepatitis B virus (HBV) is characterized by extensive genetic diversity, including low-frequency variants that contribute to disease progression. We aimed to characterize low-frequency variants and evaluate their potential clinical impact. (2) Methods: We utilized 104 HBV near-full-length sequences generated using next-generation sequencing (NGS) from people living with HIV (PLHIV). We used an in-house bioinformatics suite (HBVgenomeR v5.9.7) to filter for low-frequency variants (5–50%), which were compared to escape and drug resistance mutations (DRMs) and hepatocellular carcinoma (HCC)-associated mutations reported at the consensus level. Unclassified variants were characterized by HBV open reading frames (ORFs) to determine mutation frequency per genomic region. (3) Results: A total of six escape mutations were detected in 8/104 (7.7%) sequences, with _surface_N131T being the most prevalent (5/8). We also observed six DRMs in 30/104 (28.8%), with _rt_V173L being the most prevalent (21/30). Truncation mutations were also observed with _rt_A181T/_s_W172* and _rt_M204I/_s_W196L being the most prevalent. A total of 8/104 (7.7%) sequences had four variants associated with HCC. The _x_P46S was the highest observed HCC-associated mutation at 5/8. We report 1152 unique uncharacterized variants across all ORFs, and these were found in 94/104 (90.4%) sequences. The RNaseH domain had the highest burden (330/1152, 28.6%). (4) Conclusions: Deep sequencing results identified clinically significant mutations, including those below the 20% detection limit of traditional sequencing, that would go unreported. This highlights the possible underreporting of mutational burden in people living with HBV/HIV, indicating the importance of deep sequencing to aid in HBV/HIV understanding and management.

## 1. Introduction

The hepatitis B virus (HBV) is a highly variable DNA virus due to its unique replicative strategy involving a reverse transcriptase (RT) that replicates via an RNA intermediate, the pregenomic RNA (pgRNA) and lacks proofreading activity [1]. Due to the lack of proofreading activity in its RT domain, HBV exhibits a high mutation rate which leads to significant genetic variability [1,2]. This variability is characterized by different forms, including genotypes, sub-genotypes, quasispecies, and mutations across the viral genome [3]. The HBV genome consists of four overlapping open reading frames (ORFs) which further contribute to its complex mutation landscape, with alterations potentially occurring in any genomic region, thereby influencing disease progression and management outcomes [4,5]. The surface ORF is completely overlapped by the polymerase ORF, therefore any changes that are related to antiviral resistance in the polymerase may also occur in the surface ORF [6].

A critical component of this genetic heterogeneity is the presence of low-frequency variants which have the potential to contribute to hepatitis B disease progression as they can influence treatment outcomes, vaccine response, and HBV diagnostic failure [7,8]. While these variants represent a critical component of the viral genome pool, their role is often underestimated due to limitations in detection. Traditional sequencing methods, which are commonly used in low- and middle-income settings, such as the Sanger sequencing platform, have limited sensitivity and typically fail to detect variants comprising less than 20% of the viral quasispecies [9]. As a result, the clinical implications of these low-frequency variants remain unanswered.

Recent advances in next-generation sequencing (NGS) have allowed for superior sensitivity in detecting low-frequency variants and its deep sequencing capacity provides detailed information about the genetic variation of a virus [10]. Detecting low-frequency HBV variants is therefore important as it will provide deeper insights into our understanding of intra-host viral diversity and the emergence of clinically relevant mutations. Although present at low frequencies, these variants may be selected over time under certain pressures, potentially influencing HBV clinical outcomes, as it has been described for HIV [11]. NGS enables the identification of co-infections, rare mutations, and immune or drug escape variants with much greater precision compared to Sanger sequencing methods [9,12]. This enhanced sensitivity has significant potential for informing clinical management strategies and improving outcomes, as it enables the detection of low-frequency variants, as shown by previous studies [13,14]. NGS studies have further revealed that approximately 68% of chronic hepatitis B patients harbor low-frequency drug-resistant mutations (DRMs) (mutation frequency 0.1–5%) before treatment, and these cryptic variants may pose potential risks of treatment failure [15].

Previous HBV studies in Botswana have reported consensus-level mutations (>50%), but the burden and clinical relevance of low-frequency HBV minority variants among people living with HIV (PLHIV) remain poorly characterized [16,17,18,19,20,21]. This indicates an ongoing viral evolution emphasizing the importance of also studying the minority variants, as these could serve as potential reservoirs of emerging mutations over time. We therefore aimed to characterize low-frequency HBV variants (5–50%) associated with drug resistance (DRMs) and escape mutations in PLHIV using deep sequencing. This threshold range was selected to capture variants below the 20% detection limit of traditional sequencing, while excluding variants above 50% which represent the dominant consensus population.

## 2. Materials and Methods

### 2.1. Study Population

This retrospective, cross-sectional, sequence-based analysis study was done using previously generated near-full-length HBV sequences from the Botswana Combination Prevention Project (BCPP) study [16]. The BCPP was a pair-matched, community-randomized trial that tested whether a strategy of treatment and interventions to prevent HIV infection would reduce the population-level cumulative incidence of HIV infection over 29 months [22]. This study enrolled 12,610 consenting adults aged 16–64 years who were Botswana citizens or spouses of citizens between the years 2013 and 2018 [23]. The BCPP has been the largest cohort in Botswana, with over 3000 individuals screened for HBV to date [16,18].

### 2.2. Ethical Approval

Approval for the study was sought and granted by the Health Research Development Committee (HRDC) at the Botswana Ministry of Health HPRD: 6/14/1, 8 January 2025.

### 2.3. HBV Sequencing and Bioinformatics Analysis

A total of 104 near-full-length sequences, each obtained from a unique participant, were generated using the GridION platform (Oxford Nanopore Technologies, Oxford, UK). The library preparation used followed previously described and modified protocols [24,25]. The full sequencing protocol was described elsewhere [21]. These sequences represented genotypes A, D, and E which were used for minority variants analysis. To analyze for minority variants, we used an enhanced in-house bioinformatics suite, HBVgenomeR v5.9.7 (https://genomer-hbv.bhp.org.bw). This bioinformatics tool utilizes built-in HBV references for alignment of reads, as it has an extensive reference database. One of HBVgenomeR’s output files is an annotated variant file which contains columns for nucleotide substitutions and their corresponding amino acid changes, variant depth of coverage and their percentages, and the corresponding ORF. We filtered for minority variants by the number of reads, keeping only those ≥5, and this was followed by the variant percentage, keeping a threshold of 5–50% with anything <5% deemed a possible sequencing error, and anything >50% a consensus variant.

After filtering, the protein variants retained were grouped according to their respective ORF annotation as provided by HBVgenomer. Variants within the polymerase were compared to previously published DRMs at the consensus level, whereas those annotated in the surface region were compared to previously reported escape-associated mutations [16,21]. Variants were also compared with previously reported truncation mutations and those associated with hepatocellular carcinoma (HCC) [26,27,28,29]. All other variants were deemed unclassified variants, and their distribution was characterized by ORF.

### 2.4. Statistical Analysis

Summary tables were generated for all characterized DRMs and escape mutations, and these were participant-based. For escape mutations, >1 mutation observed per participant was reported, together with the frequency of participants harboring each combination, and their variant percentage and age were summarized as medians with interquartile range (IQR, Q1–Q3). For observed DRMs, their combination patterns were similarly reported, together with the median HBV viral load (HBV VL) and IQR (Q1–Q3). HBV VL was further used to compare between participants with and without DRMs using Wilcoxon rank-sum test. All statistical analysis was done using R version 4.6.0 (R Foundation for Statistical Computing, Vienna, Austria), and *p*-values < 0.05 were deemed statistically significant.

## 3. Results

### 3.1. Participants’ Clinical Characteristics

Table 1 summarizes the clinical characteristics of the participants whose HBV sequences were used in this study. A total of 104 participants’ HBV sequences were used in this study. Most participants were hepatitis B surface antigen (HBsAg)-positive (85.6%), with genotype A being the most prevalent (Table 1). Majority of the participants, 60.5% were on 3TC or TDF containing HIV treatment and 37.5% had no recorded regimen, including the 20.2% who were ART-naïve. Only 23.6% of the participants had HBV viral loads ≥2000.

### 3.2. Escape Mutations

**Figure 1:**
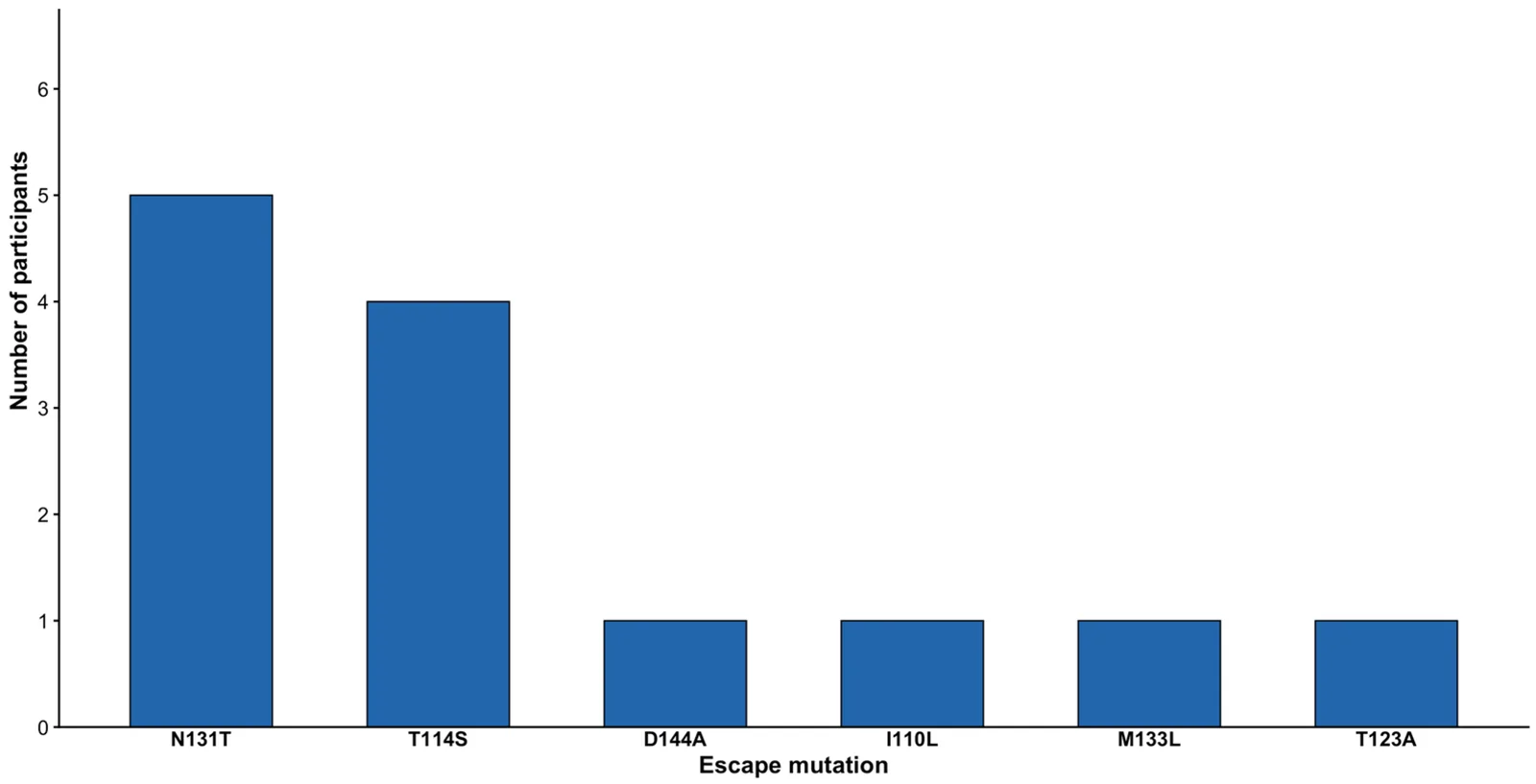
Bar graph showing overall escape mutations (*n* = 6) observed at low-frequency mutations.

A total of six escape mutations were detected in 8/104 (7.69%) sequences (Figure 1). The _surface_N131T was the most prevalent, found in 5/8 (62.5%) participants. The _surface_T114S was also prevalent at 50%. From the escape mutations detected, some participants had more than one mutation, and the N131T/T114S was the most frequent combination observed, with the other combination only appearing once (Table 2). A full description of the observed escape mutations is provided (Table S1).

### 3.3. Drug Resistance Mutations

**Figure 2:**
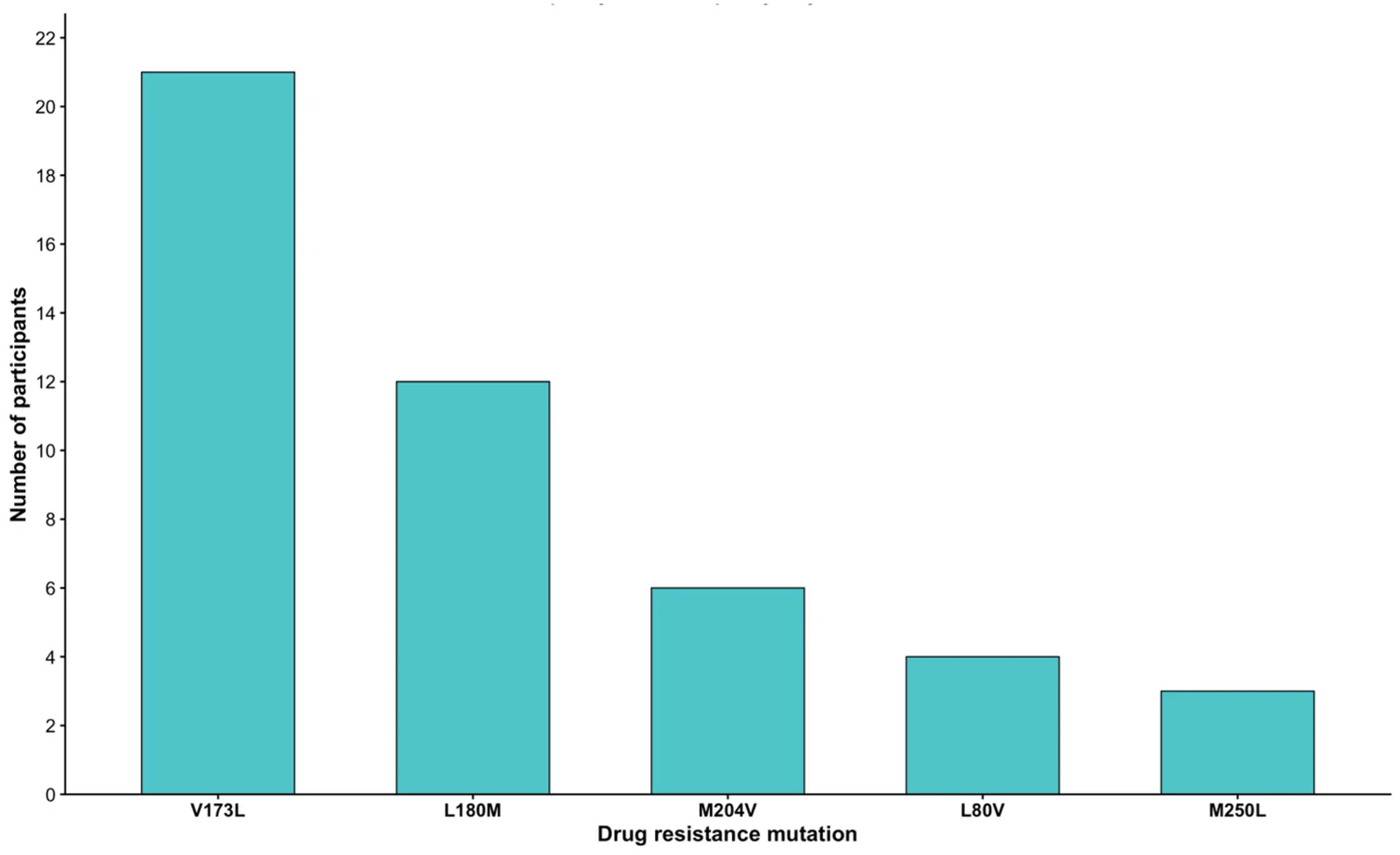
Bar graph showing overall drug resistance mutations (DRMs) observed at low-frequency mutations.

Figure 2 shows that six DRMs were detected in 30/104 (28.8%) participants. The V173L was the highly prevalent 21/30 (70%) mutation and it is associated with resistance to lamivudine (3TC). The L180M and L80V were also detected and these are variants linked with resistance to 3TC, telbivudine, and entecavir. M204V is another DRM which was observed and it is one of the most common primary resistance mutations which decreases the susceptibility to nucleos(t)ide analogs (NAs), mainly 3TC.

**Figure 3:**
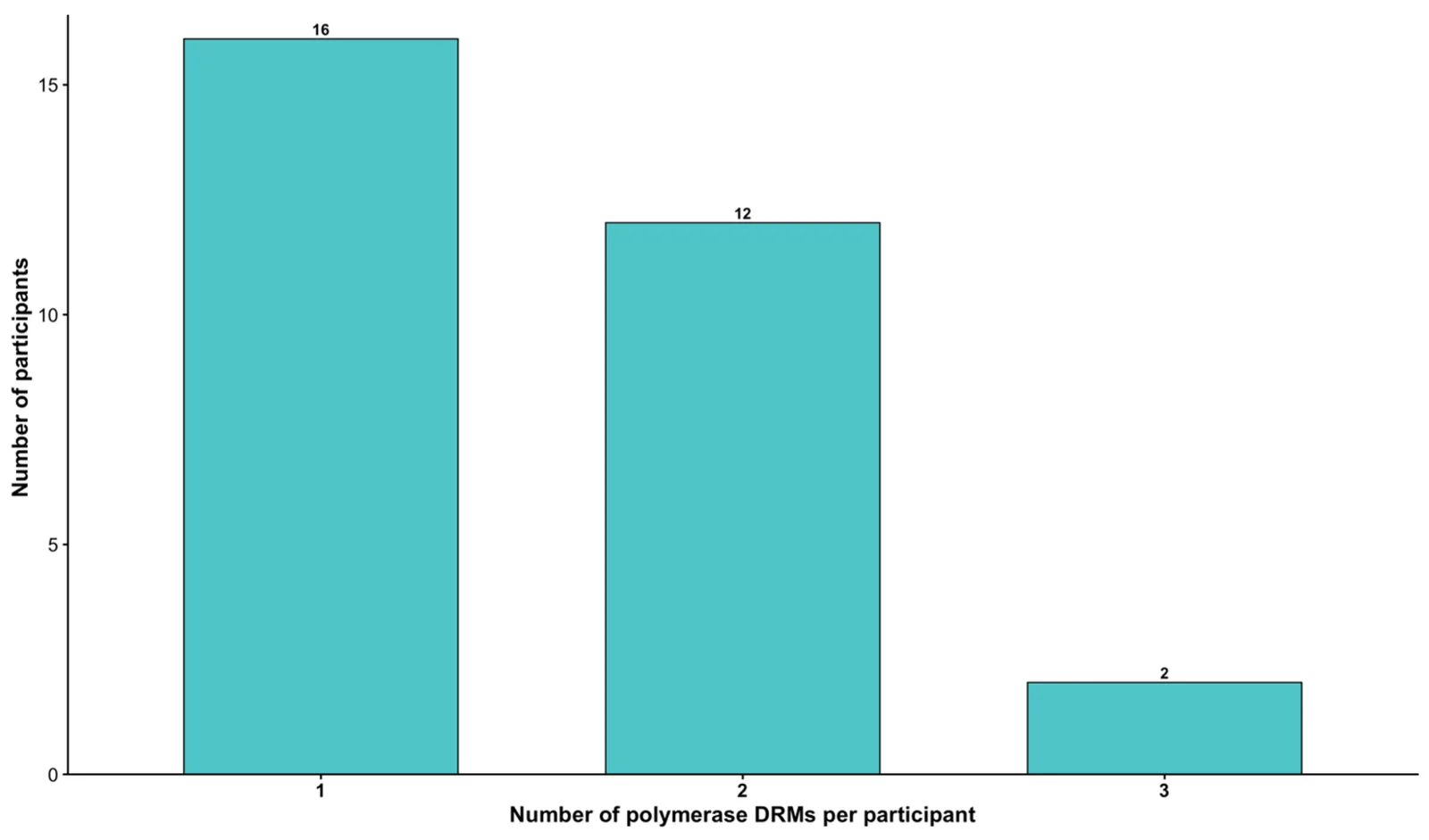
Bar graph showing distribution of DRM burden per participant.

Drug resistance-associated low-frequency variants were detected in various combinations among participants, although most harbored only a single mutation (Figure 3). The L180M/V173L combination was the most prevalent among participants receiving ART. Two participants who had previously received TDF had triple mutations, L180M/M204V/V173L and L180M/L80V/V173L. Individual mutations were also identified, with V173L detected in 30% (9/30) of participants, followed by L180M in 13% (4/30), and M204V in 6.7% (2/30) (Table 3).

**Figure 4:**
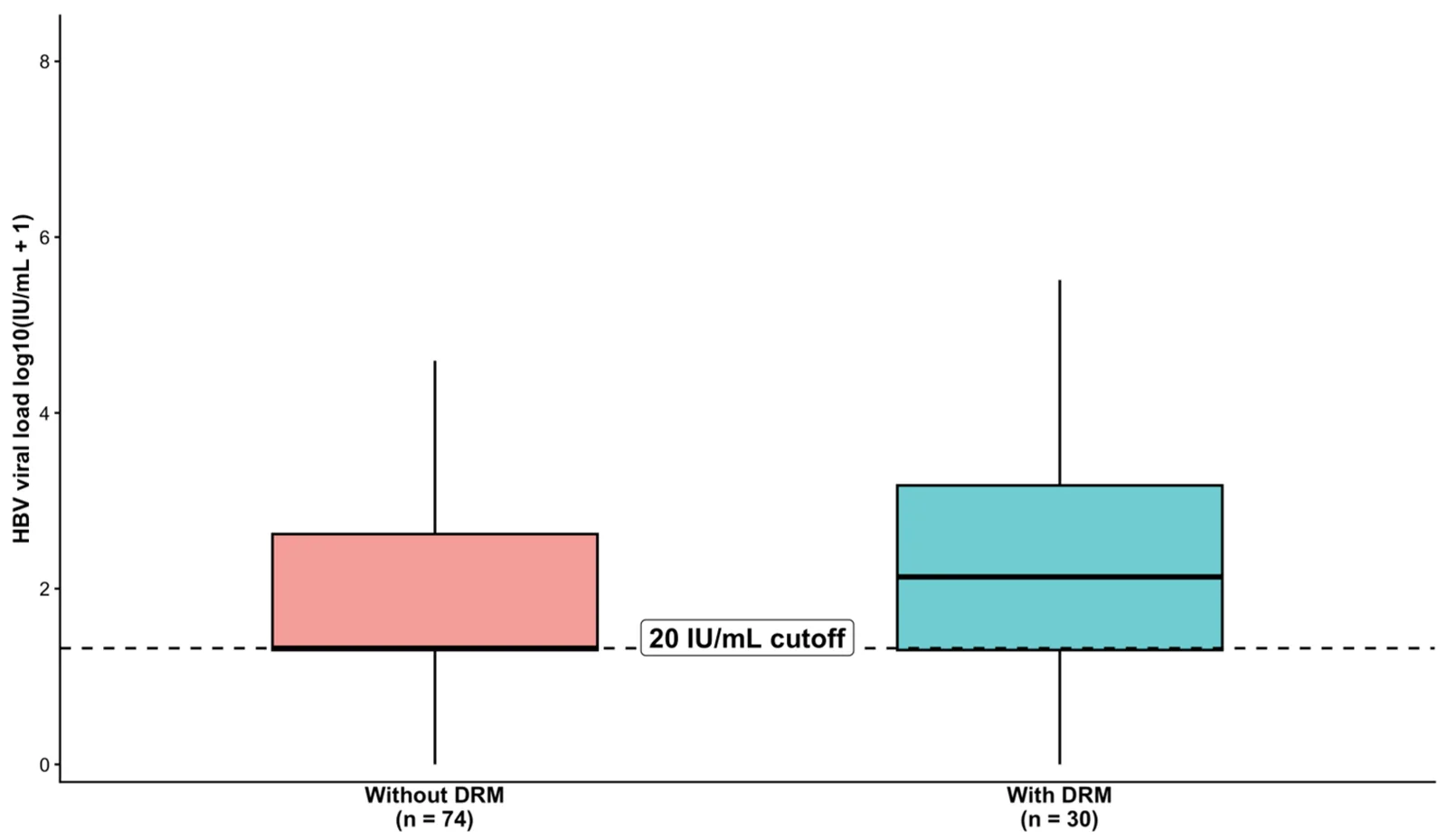
Box plot showing HBV viral load distribution among participants with and without DRMs.

In Figure 4, we assessed whether having DRMs at minority level was associated with HBV VL, by comparing participants with and without DRMs using a 20 IU/mL assay quantification cutoff. The dashed horizontal line indicates the quantification cutoff of 20IU/mL (Figure 4). Although participants with DRMs had a higher median HBV VL (142 IU/mL) than those without DRMs (20 IU/mL), the difference was not statistically significant, Wilcoxon rank-sum test, *p* = 0.25.

### 3.4. Truncation Mutations

**Figure 5:**
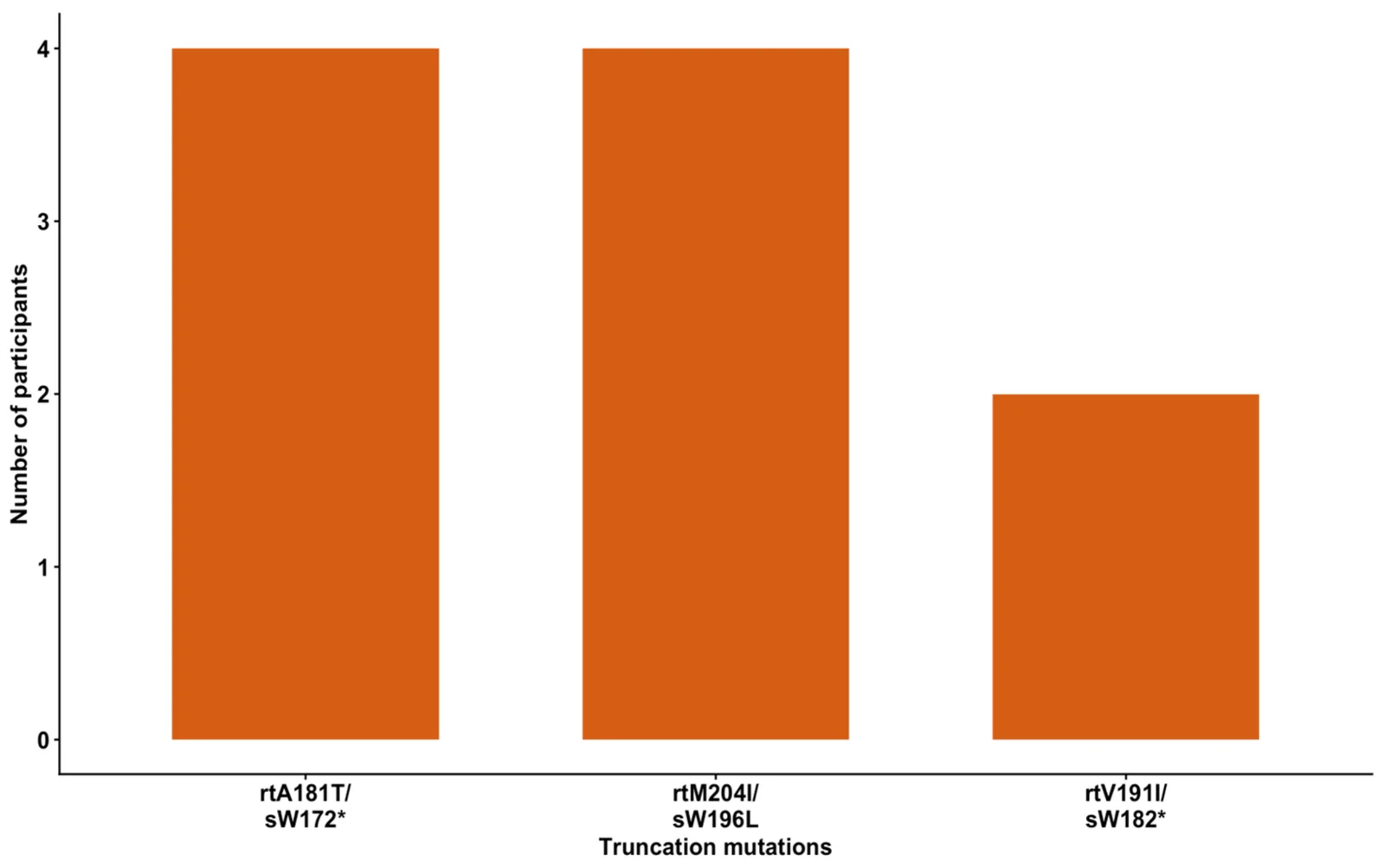
Bar graph showing HBV truncation mutations per participant.

Figure 5 shows the HBV truncation mutations observed among the participants, highlighting that 10/104 (9.6%) had these mutations. The _rt_A181T/_s_W172* and _rt_M204I/_s_W196L were both the most prevalent with four participants each harboring these combinations. The _rt_V191I/_s_W182* had the least number of participants with these mutations (Figure 5).

### 3.5. Hepatocellular Carcinoma Mutations

**Figure 6:**
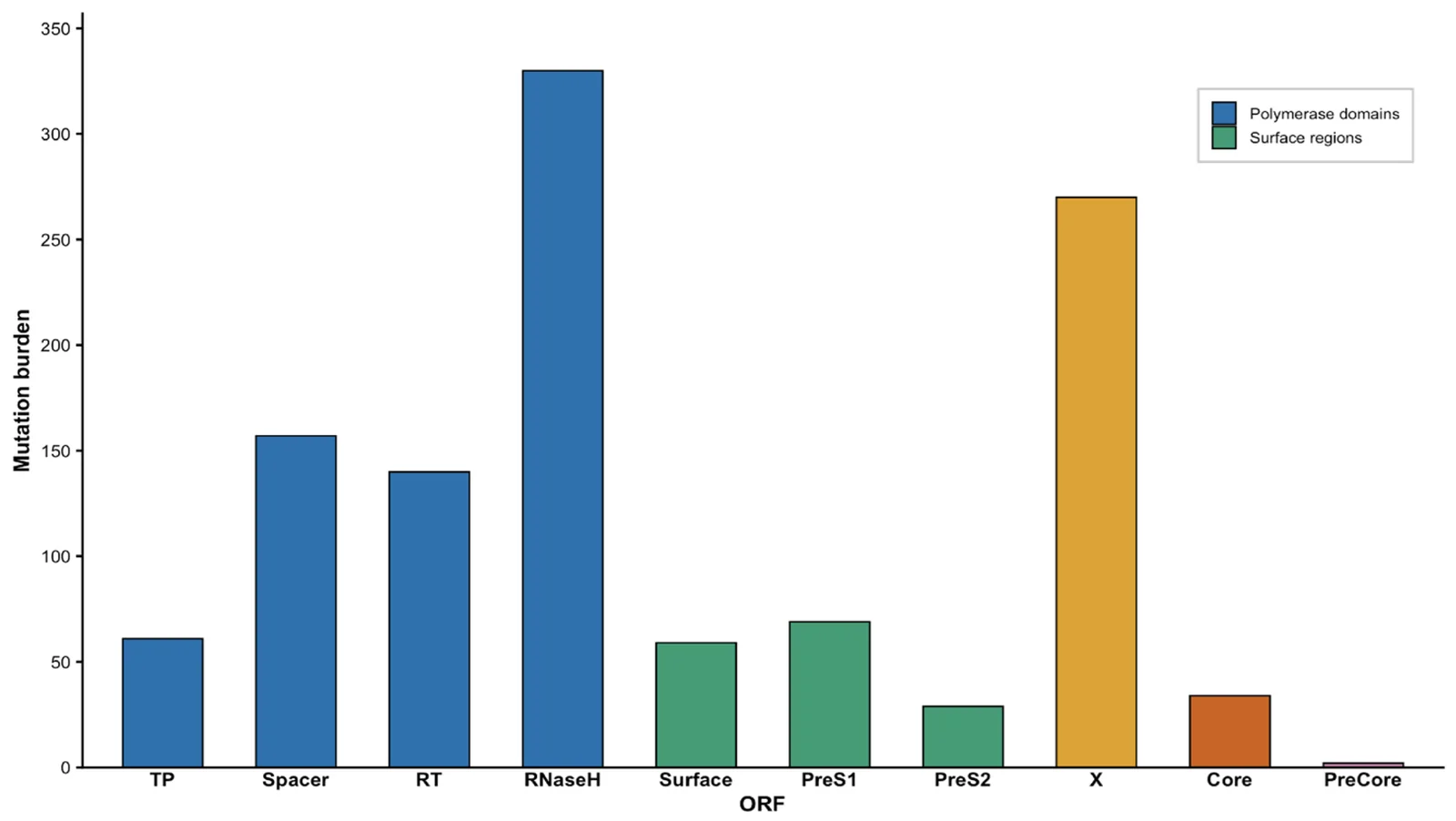
Bar graph showing the burden of uncharacterized variants in HBV ORFs observed at low-frequency mutations.

Minority variants which were not classified as either escape or drug resistance mutations were compared to those previously reported to be associated with HCC, and a total of four of these variants were observed, _surface_W172*, _x_T36A, _x_G50R, _x_P46S (Table 4). These were found in 8/104 (7.7%) sequences. All unclassified variants (*n* = 1152) were then characterized by their different ORFs, and the RNaseH domain of the polymerase ORF harbored the highest number of unclassified variants followed by the X ORF. The PreCore had the least burden of all ORFs, as shown in Figure 6. The top 10 variants in each ORF have been provided (Table S2).

## 4. Discussion

Deep sequencing identified escape mutations which have been previously reported at the consensus level by prior HBV studies in Botswana [16,18,32]. Some vaccine escape mutations that we observed in our study are the _surface_N131T, and _surface_T123A and these are a concern as they have been found to occur as a result of selection pressure from vaccination [33]. Botswana adopted the World Health Organization (WHO) recommendation in 2000 to administer a birth dose HBV vaccine to prevent perinatal and early horizontal HBV transmission [34,35]. A total of four participants who had more than a single escape mutation also had a vaccine escape mutation (VEM), but their age indicates that they were likely not vaccinated. This highlights the possibility of VEMs emerging spontaneously due to the replication mechanism of the virus.

The _surface_N131T was the most prevalent, found in 5/8 (62.5%) participants with escape-associated mutations. The _surface_M133L mutation is another escape mutation that we observed. It has been previously reported in 20% of people with occult hepatitis B virus (OBI) in southern China and it was noted to affect HBsAg detection in the local genotype B blood donors with OBI [36]. Some participants harbored more than one mutation, with N131T/T114S being the most frequent combination observed, and these two have been previously linked to immune escape, potential to decrease the effect of hepatitis B vaccination in vaccine recipients, as well as immunosuppression, HBV reactivation and impaired virion secretion, at consensus level [37,38,39,40]. The escape-associated variants we detected at minority levels have been previously associated with diagnostic escape, evasion of host immune responses, persistent infection, reduced vaccine efficacy, and hepatic damage at consensus level [37,41,42]. However, their clinical significance was not determined in this study, but our findings highlight the need for identifying and characterizing clinical significance of these escape-associated variants at minority level.

We detected DRMs in the minority variant population, and these were found in various combinations within the participants. The detected DRMs were found in participants with varying HBV VLs consistent with what was reported at consensus level [21]. A number of the detected DRMs are associated with 3TC resistance, and 3TC is a low-genetic-barrier drug for HBV [21,43]. These include V173L (30%), L180M (13%), and M204V (6.7%), and these are noted to confer resistance to 3TC as well as enhance viral replication [21]. These DRMs have been previously reported at consensus level, although the prevalences observed differ, with M204V prevalence having decreased in our study and the V173L prevalence increasing [21]. The V173L, despite being the most prevalent in our study, has been noted to previously occur at relatively low frequencies and is usually found in immunocompromised 3TC-resistant variants and has been observed as a compensatory mutation in patients with L180M/M204V mutants [44]. The double combination of L180M/V173L was observed without the presence of M204V, which is very interesting, as these have often only been co-selected with the resistance mutations at RT position 204 [45]. The triple combination of L180M/M204V/V173L was also observed in our study and it is primarily associated with 3TC resistance [46]. However, this triple combination was found in a participant that was on TDF, and this may be because earlier first-line regimens in Botswana included 3TC, and a number of participants in our study had been on 3TC-containing regimens, therefore highlighting the possibility of why the participant harbored variants associated with 3TC resistance [21]. It has been noted that 3TC HBV mutations tend to be associated with lower VLs compared to wild-type strains, but out of 10 participants in Table 3 who were shown to be on 3TC, five of them had very high VL, which differs from what has been previously reported [46]. The observed minority mutations of HBV in these participants ranged between 5.4 and 41.2%, with a median frequency of 14.8% (Q1–Q3: 8.3–32.0%). Even though some mutations were present at higher minority frequencies, they do not represent the dominant viral population; therefore, the contribution of these 3TC resistance-associated mutations to the high HBV VL observed remains unclear.

In this study, we were able to detect truncation mutations due to the overlap between the surface and polymerase ORFs. As a result, mutations in the polymerase ORF can lead to corresponding surface ORF mutations [29]. Detecting these mutations is important as these have been theoretically associated with HCC, although clinical evidence remains inconclusive regarding their direct role in accelerating HCC development [26,29]. The _rt_A181T/_s_W172*, _rt_M204I/_s_W196* and _rt_V191I/_s_W182* are truncated mutations that have been found in high frequencies and decrease the antiviral effect and have oncogenic potential and these are the same mutations that we have observed [47]. The _rt_M204I mutation leads to either prematurely truncated small surface protein _s_W196*(Stop) mutation or substitution mutation as _s_W196L at the corresponding overlapping region, of which in our study, we observed the _s_W196L [48].

Other than the escape mutations and DRMs, we also observed some mutations that have been previously noted to be associated with HCC, in 8/104 sequences. The _x_P46S was the highest observed HCC-associated variant at 5/8 (62.5%). It is important to have observed these HCC-related mutations as these could promote HCC pathogenesis and liver disease progression [49]. Their detection in clinical practice could be helpful for defining better preventive and therapeutic strategies and, moreover, predicting the progression of liver disease [49].

A number of uncharacterized minority variants were observed in our sequences at different ORFs. This observation may be due to the low fidelity of the polymerase, the high replication rate and the overlapping ORFs, leading to mutations occurring throughout the genome [1]. The high burden of uncharacterized variants observed in our study highlights the importance of tracking these variants to better understand viral pathogenesis. As the RNaseH domain had the highest burden, some of the mutations observed and not characterized can probably affect the replication capacity of HBV, as RNaseH plays the role of degrading the viral ribonucleic acid (RNA) after it has been copied into deoxyribonucleic acid (DNA) by the RT to permit synthesis of the second DNA strand [50]. Following the polymerase region, the X ORF (HBx) also showed an increased mutational burden. It has been previously reported that mutations in HBx are frequently found in patients exhibiting different manifestations of HBV-associated liver complications and can be useful in predicting the clinical outcome of HBV-infected patients, and they may serve as early markers of a high risk of developing HCC [51]. The surface ORF also showed a relatively high mutation burden. Mutations found in the region known as the “a” determinant of the HBsAg, located within the major hydrophilic region (MHR) of the surface gene, usually modify the antigenicity of the protein, impairing virion secretion and HBsAg formation [52]. Some of the uncharacterized variants are located in the MHR, and these may possibly lead to vaccine escape and diagnostics failures. Association of mutations with HBV genotype was not done as 94% of the sequences were genotype A. The absence of fibrosis staging and other markers of liver disease progression limited our ability to evaluate the clinical significance of the detected mutations and their association with disease progression.

The study’s main limitation is that all participants were PLHIV, with no HBV mono-infected comparison group, and this limits the generalizability of our findings to people with HBV mono-infection. Compared with mono-infection, HBV/HIV coinfection accelerates HBV-related liver damage, with faster liver fibrosis progression, and greater risk of end-stage liver disease (ESLD) and HCC compared to HBV mono-infected patients [53]. HIV-associated immune dysfunction may also reduce immune control of HBV, which can also allow for minority variants to persist [53]. Exposure to ART regimens which contain HBV-active regimens, such as the 3TC-containing treatment, may allow for selective pressure which may favor drug resistance mutations as these have a low barrier to resistance [21,54]. However, as an HBV mono-infection group was not included, the effects of HIV-related immunosuppression and possible side effects of ART could not be determined. Furthermore, the cross-sectional retrospective nature of this study resulted in the absence of important clinical data to determine whether participants harboring HBV minority variants previously associated with drug resistance, immune escape, truncation, or HCC experienced increased rates of treatment failure, progressive liver disease, or HCC. Further longitudinal studies incorporating clinical outcomes are needed to establish the clinical significance of these minority variants.

## 5. Conclusions

HBV deep sequencing revealed extensive genetic diversity of HBV minority variants, with escape mutations detected in 7.7% participants, DRMs in 28.8%, and HCC-associated mutations in 7.7%. We also observed HBV truncation mutations in 9.6% participants. A number of minority variants observed were not characterized previously, meaning their functional relevance is still unknown. This study has added to the body of knowledge of HBV minority variants, highlighting the underreporting of the actual mutational burden of HBV which may affect treatment and management. This study emphasizes the importance of deep sequencing to aid in HBV/HIV understanding and management.

## Figures and Tables

**Table 1:** Clinical characteristics of the participants.

Characteristics	Number (%) *n* = 104
HBV type	
HBsAg+	89 (85.6)
OBI	15 (14.4)
HBV genotypes	
A	98 (94.2)
D	3 (2.9)
E	3 (2.9)
HBV viral load	
HBsAg+, *n* = 89	
<2000	43 (48.3)
≥2000	21 (23.6)
TND	25 (28.1)
OBI, *n* = 15	
<20	14 (93.3)
≥20	1 (6.7)
ART status	
Naïve	21 (20.2)
On ART	83 (79.8)
ART regimen	
3TC-containing regimen	30 (28.8)
TDF-containing regimen	33 (31.7)
No 3TC/TDF-containing regimen	2 (1.9)
Unknown	39 (37.5)

Abbreviations: HBV, hepatitis B virus; HBsAg, hepatitis B surface antigen; OBI, occult hepatitis B virus; TND, target not detected; ART, antiretroviral therapy; 3TC, lamivudine; TDF, tenofovir disoproxil fumarate.

**Table 2:** Combination of escape mutations observed in participants.

>1 Mutation Combinations	Frequency	% of Reads, Median (Q1–Q3)	Age, Median (Q1–Q3)
N131T/T114S	3	N131T: 25.9 (21.5–27.2)	41 (35.9–49)
		T114S: 21.9 (17.6–26.6)	
D144A/N131T/T114S	1	37.2/5.1/6.8	50.1

**Table 3:** Combination of DRMs observed in participants with low-frequency minority DRMs.

Combination	Mutations	ARV Status	ART Regimen	Frequency	HBV VL (IU/mL), Median (Q1–Q3)
Single Mutations	M204V	All on ART	No data	2	2847.5 (1557.8–4137.2)
	V173L	All on ART	3TC	2 2	8.5 × 10^7^ (42,500,000–127,500,000) 85,000,026 (42,500,039–127,500,013)
			No data TDF	2 3	NA 247 (123.5–376)
	L180M	All on ART	3TC TDF	2 2	28 1143 19 NA
	L80V	All on ART	No data	1	356
Double Mutations	L80V/M250L	All on ART	3TC	1	1.7 × 10^8^
	L80V/V173L	All on ART	3TC	1	6532
	M204V/V173L	All on ART	No data	1 1	85 3298
	M250L/V173L	All on ART	3TC TDF	1 1	NA 94
	L180M/V173L	ART naive All on ART	No data TDF No data	1 1 3	19 NA 1633 (826–163,531)
	L180M/M204V	All on ART	3TC	1	99
Triple Mutations	L180M/M204V/V173L	ART naive	TDF	1	184
	L180M/L80V/V173L	ART naive	TDF	1	481

Abbreviations: ART, antiretroviral therapy; 3TC, lamivudine; TDF, tenofovir disoproxil fumarate.

**Table 4:** Minority variants associated with HCC.

Mutations	Clinical Impact	Reference
_surface_W172* (a.a) G670A (nt)	Generates a stop codon in the HBsAg, impairing the secretion of HBsAg. Increases the risk of HCC.	[27,30]
_x_T36A (a.a) A1479G (nt)	It increases the viral genome integration in the host cell.	[27,31]
_x_G50R (a.a) G1521A (nt)	High association with HCC	[27,31]
_x_P46S (a.a) C1509T (nt)	High association with HCC	[28]

Abbreviations: a.a, amino acid; nt, nucleotide; HBsAg, hepatitis B surface antigen; HCC, hepatocellular carcinoma.

## Data Availability

The data that was analyzed and generated in the study is not available online due to institutional policies and patient confidentiality. The data generated in this study is available upon request from the corresponding author.

## Supplementary Materials

The following supporting information can be downloaded at: https://www.mdpi.com/article/10.3390/v18080904/s1, Table S1: Escape mutations observed and their clinical impact; Table S2: Distribution of the most frequent uncharacterized variants across HBV ORFs and polymerase domains. References [16,38,39,40,55,56,57,58,59] are cited in the Supplementary Materials.

## Abbreviations

The following abbreviations are used in this manuscript:

HBV	Hepatitis B Virus
NGS	Next-generation sequencing (NGS)
PLHIV	People living with HIV
DRM	Drug resistance mutations
HCC	Hepatocellular carcinoma
ORF	Open reading frames
RT	Reverse transcriptase
pgRNA	pregenomic RNA
BCPP	Botswana Combination Prevention Project
HRDC	Health Research Development Committee
VL	Viral Load
NA	Nucleos(t)ide analogs
VEMs	Vaccine escape mutation
OBI	Occult hepatitis B virus
MHR	Major hydrophilic region
HBsAg	Hepatitis B surface antigen
a.a	Amino acid
nt	Nucleotide
DNA	Deoxyribonucleic acid
RNA	Ribonucleic acid

## References

[1] Caligiuri P., Cerruti R., Icardi G., Bruzzone B. (2016). Overview of hepatitis B virus mutations and their implications in the management of infection. World J. Gastroenterol..

[2] Block T.M., Guo H., Guo J.T. (2007). Molecular virology of hepatitis B virus for clinicians. Clin. Liver Dis..

[3] Zhang Z.H., Wu C.C., Chen X.W., Li X., Li J., Lu M.J. (2016). Genetic variation of hepatitis B virus and its significance for pathogenesis. World J. Gastroenterol..

[4] Gao S., Duan Z.P., Coffin C.S. (2015). Clinical relevance of hepatitis B virus variants. World J. Hepatol..

[5] Osoga J., Ochwoto M., Tuitoek G., Ogonda L. (2026). Genetic Diversity of Hepatitis B Virus Genomes Isolated from Patients Attending Health Facilities in HBV-Endemic Regions in Kenya. LabMed.

[6] Arikan A., Sayan M., Sanlidag T., Suer K., Akcali S., Guvenir M. (2019). Evaluation of the pol/S Gene Overlapping Mutations in Chronic Hepatitis B Patients in Northern Cyprus. Pol. J. Microbiol..

[7] Mlewa M., Henerico S., Nyawale H.A., Mangowi I., Shangali A.R., Manisha A.M., Kisanga F., Kidenya B.R., Jaka H., Kilonzo S.B. (2025). The pattern change of hepatitis B virus genetic diversity in Northwestern Tanzania. Sci. Rep..

[8] Ma Q., Wang Y. (2012). Comprehensive analysis of the prevalence of hepatitis B virus escape mutations in the major hydrophilic region of surface antigen. J. Med. Virol..

[9] Yano Y., Azuma T., Hayashi Y. (2015). Variations and mutations in the hepatitis B virus genome and their associations with clinical characteristics. World J. Hepatol..

[10] Satam H., Joshi K., Mangrolia U., Waghoo S., Zaidi G., Rawool S., Thakare R.P., Banday S., Mishra A.K., Das G. (2023). Next-Generation Sequencing Technology: Current Trends and Advancements. Biology.

[11] Watson S.J., Welkers M.R., Depledge D.P., Coulter E., Breuer J.M., de Jong M.D., Kellam P. (2013). Viral population analysis and minority-variant detection using short read next-generation sequencing. Philos. Trans. R. Soc. Lond. B Biol. Sci..

[12] Hebeler-Barbosa F., Wolf I.R., Valente G.T., Mello F.C.D.A., Lampe E., Pardini M.I.M.C., Grotto R.M.T. (2020). A New Method for Next-Generation Sequencing of the Full Hepatitis B Virus Genome from A Clinical Specimen: Impact for Virus Genotyping. Microorganisms.

[13] Rodriguez C., Chevaliez S., Bensadoun P., Pawlotsky J.M. (2013). Characterization of the dynamics of hepatitis B virus resistance to adefovir by ultra-deep pyrosequencing. Hepatology.

[14] Solmone M., Vincenti D., Prosperi M.C., Bruselles A., Ippolito G., Capobianchi M.R. (2009). Use of massively parallel ultradeep pyrosequencing to characterize the genetic diversity of hepatitis B virus in drug-resistant and drug-naive patients and to detect minor variants in reverse transcriptase and hepatitis B S antigen. J. Virol..

[15] CDGenomics Hepatitis B Virus Genome and Clinical Implications. https://www.cd-genomics.com/resource-hepatitisb-virus-genome-clinal.html.

[16] Phinius B.B., Choga W.T., Anderson M., Mokomane M., Gobe I., Ratsoma T., Phakedi B., Mpebe G., Bhebhe L., Gaolathe T. (2024). Molecular Characterization of Hepatitis B Virus in People Living with HIV in Rural and Peri-Urban Communities in Botswana. Biomedicines.

[17] Anderson M., Gaseitsiwe S., Moyo S., Wessels M.J., Mohammed T., Sebunya T.K., Powell E.A., Makhema J., Blackard J.T., Marlink R. (2015). Molecular characterisation of hepatitis B virus in HIV-1 subtype C infected patients in Botswana. BMC Infect. Dis..

[18] Anderson M., Mangogola T., Phinius B.B., Mpebe G., Aimakhu C.O., Choga W.T., Phakedi B., Bhebhe L.N., Ditshwanelo D., Baruti K. (2024). Hepatitis B Virus Prevalence among HIV-Uninfected People Living in Rural and Peri-Urban Areas in Botswana. Microorganisms.

[19] Tsayang C.D., Schanzer E., Phinius B.B., Mulenga G., Molebatsi K., Lechiile K., Bhebhe L., Ratsoma T., Mpebe G.G.A., Mulenga F. (2026). High Prevalence of Hepatitis B Virus Infection Among People Living with Advanced HIV Disease in Botswana. Biomedicines.

[20] Choga W.T., Anderson M., Zumbika E., Moyo S., Mbangiwa T., Phinius B.B., Melamu P., Kayembe M.K., Kasvosve I., Sebunya T.K. (2019). Molecular characterization of hepatitis B virus in blood donors in Botswana. Virus Genes.

[21] Phinius B.B., Anderson M., Gobe I., Mokomane M., Choga W.T., Phakedi B., Ratsoma T., Mpebe G., Makhema J., Shapiro R. (2024). High Prevalence of Hepatitis B Virus Drug Resistance Mutations to Lamivudine among People with HIV/HBV Coinfection in Rural and Peri-Urban Communities in Botswana. Viruses.

[22] Makhema J., Wirth K.E., Pretorius Holme M., Gaolathe T., Mmalane M., Kadima E., Chakalisa U., Bennett K., Leidner J., Manyake K. (2019). Universal Testing, Expanded Treatment, and Incidence of HIV Infection in Botswana. N. Engl. J. Med..

[23] Gaolathe T., Wirth K.E., Holme M.P., Makhema J., Moyo S., Chakalisa U., Yankinda E.K., Lei Q., Mmalane M., Novitsky V. (2016). Botswana’s progress toward achieving the 2020 UNAIDS 90-90-90 antiretroviral therapy and virological suppression goals: A population-based survey. Lancet HIV.

[24] Phinius B.B., Anderson M., Mokomane M., Gobe I., Choga W.T., Ratsoma T., Phakedi B., Mpebe G., Ditshwanelo D., Musonda R. (2023). Atypical Hepatitis B Virus Serology Profile-Hepatitis B Surface Antigen-Positive/Hepatitis B Core Antibody-Negative-In Hepatitis B Virus/HIV Coinfected Individuals in Botswana. Viruses.

[25] Choga W., Yeshnee M., Chabuka L., Maponga T., Tshiabuila D., San J.E., Tegally H., Moir M., Pillay S., Lessells R. (2023). Complete Hepatitis B Virus Sequencing Using an ONT-Based Next-Generation Sequencing Protocol v1.

[26] Wang M.-L., Tang H. (2016). Nucleos(t)ide analogues causes HBV S gene mutations and carcinogenesis. Hepatobiliary Pancreat. Dis. Int..

[27] Shoraka S., Hosseinian S.M., Hasibi A., Ghaemi A., Mohebbi S.R. (2023). The role of hepatitis B virus genome variations in HBV-related HCC: Effects on host signaling pathways. Front. Microbiol..

[28] Belaiba Z., Ayouni K., Gdoura M., Kammoun Rebai W., Touzi H., Sadraoui A., Hammemi W., Yacoubi L., Abdelati S., Hamzaoui L. (2022). Whole genome analysis of hepatitis B virus before and during long-term therapy in chronic infected patients: Molecular characterization, impact on treatment and liver disease progression. Front. Microbiol..

[29] Yan T., Zhang Y., Zhou H., Jiang N., Wang X., Yan W., Yin J. (2025). Surface Gene Mutations of Hepatitis B Virus and Related Pathogenic Mechanisms: A Narrative Review. Viruses.

[30] Yeh C.T., Chen T., Hsu C.W., Chen Y.C., Lai M.W., Liang K.H., Chen T.C. (2011). Emergence of the rtA181T/sW172* mutant increased the risk of hepatoma occurrence in patients with lamivudine-resistant chronic hepatitis B. BMC Cancer.

[31] Tuteja A., Siddiqui A.B., Madan K., Goyal R., Shalimar, Sreenivas V., Kaur N., Panda S.K., Narayanasamy K., Subodh S. (2014). Mutation Profiling of the Hepatitis B Virus Strains Circulating in North Indian Population. PLoS ONE.

[32] Anderson M., Choga W.T., Moyo S., Bell T.G., Mbangiwa T., Phinius B.B., Bhebhe L., Sebunya T.K., Lockman S., Marlink R. (2018). Molecular Characterization of Near Full-Length Genomes of Hepatitis B Virus Isolated from Predominantly HIV Infected Individuals in Botswana. Genes.

[33] Leong J., Lin D., Nguyen M.H. (2016). Hepatitis B surface antigen escape mutations: Indications for initiation of antiviral therapy revisited. World J. Clin. Cases.

[34] Baruti K., Lentz K., Anderson M., Ajibola G., Phinius B.B., Choga W.T., Mbangiwa T., Powis K.M., Sebunya T., Blackard J.T. (2020). Hepatitis B virus prevalence and vaccine antibody titers in children HIV exposed but uninfected in Botswana. PLoS ONE.

[35] Patel P., Davis S., Tolle M., Mabikwa V., Anabwani G. (2011). Prevalence of Hepatitis B and Hepatitis C Coinfections in an Adult HIV Centre Population in Gaborone, Botswana. Am. Soc. Trop. Med. Hyg..

[36] Ye X., Liu L., Chen L., Nie X., Huang L., Ye D., Zeng J., Li T., Li B., Xu M. (2022). High-Frequency Notable HBV Mutations Identified in Blood Donors With Occult Hepatitis B Infection From Heyuan City of Southern China. Front. Immunol..

[37] King R.E., Kimotho J., Macharia R., Ndung’u F.N., Nzou S.M., Irekwa R.M. (2023). Identification and Characterization of Hepatitis B Virus Immune Escape Mutants in Kenya. Am. J. Mol. Biol..

[38] Khodadad N., Seyedian S.S., Moattari A., Biparva Haghighi S., Pirmoradi R., Abbasi S., Makvandi M. (2020). In silico functional and structural characterization of hepatitis B virus PreS/S-gene in Iranian patients infected with chronic hepatitis B virus genotype D. Heliyon.

[39] Kwei K., Tang X., Lok A.S., Sureau C., Garcia T., Li J., Wands J., Tong S. (2013). Impaired virion secretion by hepatitis B virus immune escape mutants and its rescue by wild-type envelope proteins or a second-site mutation. J. Virol..

[40] Rajput M.K. (2020). Mutations and methods of analysis of mutations in Hepatitis B virus. AIMS Microbiol..

[41] Coleman P.F. (2006). Detecting hepatitis B surface antigen mutants. Emerg. Infect. Dis..

[42] De Francesco M.A., Gargiulo F., Dello Iaco F., Zeneli L., Zaltron S., Tiecco G., Pellizzeri S., Focà E., Caruso A., Quiros-Roldan E. (2025). Immune Escape and Drug Resistance Mutations in Patients with Hepatitis B Virus Infection: Clinical and Epidemiological Implications. Life.

[43] Ghany M.G., Doo E.C. (2009). Antiviral resistance and hepatitis B therapy. Hepatology.

[44] Delaney W.E., Yang H., Westland C.E., Das K., Arnold E., Gibbs C.S., Miller M.D., Xiong S. (2003). The hepatitis B virus polymerase mutation rtV173L is selected during lamivudine therapy and enhances viral replication in vitro. J. Virol..

[45] Pallier C., Castéra L., Soulier A., Hézode C., Nordmann P., Dhumeaux D., Pawlotsky J.M. (2006). Dynamics of hepatitis B virus resistance to lamivudine. J. Virol..

[46] Sultana C., Casian M., Oprea C., Ianache I., Grancea C., Chiriac D., Ruta S. (2022). Hepatitis B Virus Genotypes and Antiviral Resistance Mutations in Romanian HIV-HBV Co-Infected Patients. Medicina.

[47] Neumann-Fraune M., Beggel B., Pfister H., Kaiser R., Verheyen J. (2013). High frequency of complex mutational patterns in lamivudine resistant hepatitis B virus isolates. J. Med. Virol..

[48] Khan S., Anwer A., Sevak J.K., Abbas Z., Fatima K., Islam M., Jehan T., Trehanpati N., Kazim S.N. (2025). Expression variation of Akt and cytokines in lamivudine-resistant HBV rare mutation that prematurely truncates the overlapping surface protein. Hum. Immunol..

[49] Zhang X., Ding H.G. (2015). Key role of hepatitis B virus mutation in chronic hepatitis B development to hepatocellular carcinoma. World J. Hepatol..

[50] Edwards T.C., Ponzar N.L., Tavis J.E. (2019). Shedding light on RNaseH: A promising target for hepatitis B virus (HBV). Expert Opin. Ther. Targets.

[51] Al-Qahtani A.A., Al-Anazi M.R., Nazir N., Ghai R., Abdo A.A., Sanai F.M., Al-Hamoudi W.K., Alswat K.A., Al-Ashgar H.I., Khan M.Q. (2017). Hepatitis B virus (HBV) X gene mutations and their association with liver disease progression in HBV-infected patients. Oncotarget.

[52] Romani S., Hosseini S.M., Mohebbi S.R., Boonstra A., Hosseini Razavi A., Sharifian A. (2018). Characterization of the “a” determinant region of the hepatitis B virus genome in Iranian patients at different clinical phases of chronic infection. Gastroenterol. Hepatol. Bed Bench.

[53] Sarmati L., Malagnino V. (2019). HBV Infection in HIV-Driven Immune Suppression. Viruses.

[54] Woo H.Y., Park J.Y., Bae S.H., Kim C.W., Jang J.Y., Tak W.Y., Kim D.J., Kim I.H., Heo J., Ahn S.H. (2020). Entecavir+tenofovir vs. lamivudine/telbivudine+adefovir in chronic hepatitis B patients with prior suboptimal response. Clin. Mol. Hepatol..

[55] Adesina O.A., Akanbi O.A., Opaleye O.O., Japhet M.O., Wang B., Oluyege A.O., Klink P., Bock C.T. (2021). Detection of Q129H Immune Escape Mutation in Apparently Healthy Hepatitis B Virus Carriers in Southwestern Nigeria. Viruses.

[56] Lazarevic I., Banko A., Miljanovic D., Cupic M. (2019). Immune-Escape Hepatitis B Virus Mutations Associated with Viral Reactivation upon Immunosuppression. Viruses.

[57] Osiowy C., Kowalec K., Giles E. (2016). Discordant diagnostic results due to a hepatitis B virus T123A HBsAg mutant. Diagn. Microbiol. Infect. Dis..

[58] Modise L., Sithebe N., Mufhandu H. (2023). Investigation of mutations in the partial sequences of the surface and polymerase genes of hepatitis B virus associated with immune escape and drug resistance in HIV-infected patients. F1000Research.

[59] Langat B.K., Ochwedo K.O., Borlang J., Osiowy C., Mutai A., Okoth F., Muge E., Andonov A., Maritim E.S. (2023). Genetic diversity, haplotype analysis, and prevalence of Hepatitis B virus MHR mutations among isolates from Kenyan blood donors. PLoS ONE.

