# Supplementary material for: Deep Sequencing of Hepatitis B Virus Reveals Clinically Relevant Low-Frequency Variants Among People Living with HIV in Botswana
Source: Viruses. 2026 Aug 17;18(8):904. doi: 10.3390/v18080904 (PMC13517587; doi:10.3390/v18080904)
Supplement: Supplementary file 1 [file viruses-18-00904-s001.zip › Table S2.pdf]

Table S2. Distribution of the most frequent uncharacterized variants across HBV ORFs and polymerase domains.

HBV ORF	Mutation (Frequency)
PreS1	K7Q (21), G35R (13), P94T (12), S6A (10), S6P (8), Q57K (5), T68P (5), I48N (4), Q54E (4), S5L (4)
PreS2	S57F (9), N48T (7), T60I (7), A22T (6), D62V (6), A50V (4), A64V (4), S65P (4), H52L (3), H52P (3)
Surface	E164D (26), G202E (15), P203L (10), P214L (8), V180A (8), W201L (8), F19V (5), I195M (5), Q101P (5), T57I (5)
X	C61R (8), D48V (8), A31S (7), G22S (7), S12A (6), Y6C (6), A62V (5), E24K (5), L5V (5), P29L (5)
Core	W62L (16), R39Q (9), N74H (8), F18I (7), G123* (7), P45S (6), F18L (5), G123R (4), T91N (3), V13A (3)
PreCore	W28* (3), G25* (1)
TP	T165I (14), T146A (6), H87Q (5), F170L (4), T68S (4), V69I (4), F117I (3), I71V (3), Q138H (3), Q77K (3)
Spacer	R34* (45), K14T (21), K14Q (14), R55C (14), R42Q (13), S101Y (12), T15A (12), S101P (11), I13S (10), I13T (8)
RT	rtR289K (17), rtY126H (13), rtN124H (12), rtI103V (9), rtA223V (7), rtN131D (7), rtW153R (7), rtG165S (6), rtI282V (6), rtT259S (6)
RNaseH	T119S (10), Y116F (8), P130R (7), S129C (6), T77A (6), P100S (5), P130L (5), P133S (5), R113H (5), V138D (5)
