# Supplementary material for: Deep Sequencing of Hepatitis B Virus Reveals Clinically Relevant Low-Frequency Variants Among People Living with HIV in Botswana
Source: Viruses. 2026 Aug 17;18(8):904. doi: 10.3390/v18080904 (PMC13517587; doi:10.3390/v18080904)
Supplement: Supplementary file 1 [file viruses-18-00904-s001.zip › Table S1.pdf]

Table S1. Escape mutations observed and their clinical impact.

Mutation	Clinical Impact	Reference
M133L<br>D144A	Immune escape	[55,56]
T123A	Diagnostic escape	[57,58]
I110L	Diagnostic and Immune escape	[59]
T114S	Mutations in this position are correlated to immunosuppression and HBV reactivation / Impaired virion secretion	[16,39,40]
N131T	Vaccine escape	[38]
